# Scientific Items

**Published:** 1878-02-20

**Authors:** 


### SCIENTIFIC ITEMS.

### MEDICAL PROGRESS IN 1877.

The London *Lancet* devotes a large portion of a recent issue to a very full summary of the advances made in medicine and surgery during the year just closed. Of these, the most important are the following: M. Paul Bert has published an extensive work on the effect of variations of pressure on the body, and he shows that the observed effects of diminished pressure are exclusively due to a diminution in the tension of the oxygen in the air, and consequent predisposition to asphyxia; while, on the contrary, increase of pressure up to three atmospheres occasions more active intra-organic changes, and when the pressure reaches five atmospheres, the oxidizing processes either cease or become modified in such a way as to be inconsistent with the maintenance of life. Guttmann, Frickler, and Oertmann have demonstrated that the absorption of oxygen is independent of the mechanical acts of respiration. Richet has determined that, when perfectly fresh, the gastric juice contains only mineral acids, but that, after standing for sometime, a kind of fermentation is set up, in which much free organic acid is formed that, on analysis, proved to be lactic acid. It is believed to be beyond doubt that lactic, as well as butyric and acetic acids, are often either introduced into the stomach or are formed in it as a product of fermentation.

*Color of the Retina.*—By far the most interesting discovery of the year in physiology is that made by Boll, that the retina possesses, in health, a peculiar red color, which is constantly being destroyed by the influence of light, and is as constantly being regenerated by the ordinary processes of nutrition. The "vision red," or "erythopsin," as its discoverer names it, attains its maximum after a night's rest and sleep, or when an animal has been kept for some hours in darkness; it is soluble in solutions of the biliary acids and in glycerin, and probably plays a part in the production of the red reflection from the fundus of the eye seen on ophthalmoscopic examination, as well as in all probability in the ordinary acts of vision.

*Etiology of Infectious Diseases.*—The most important progress in the department of pathology is that toward the establishment and diffusion of the opinion that minute organisms are concerned in the progress of acute infectious diseases. Chaureau has shown that the horse is peculiarly receptive of the vaccine virus, and is capable of reproducing it in remarkable purity and force.—*Scientific American.*

---

Electric Light is in its infancy yet. So far, it has only been found advantageous for lighting large buildings, like public halls, railway depots, machine shops, etc. It has not yet been adapted to the requirements of private houses. The modern appliances for producing electricity, and transforming it into light, cannot be intelligibly described without drawings and illustrations.—*Druggists' Circular.*

---

### LIQUEFACTION OF GASES.

Professor Tyndall received a telegram from Professor Pictect, of Geneva, that he had succeeded in liquefying oxygen by means of extreme cold and pressure. Carbonic acid gas has been both liquefied and solidified. Both hydrogen and nitrogen have been liquefied, and on Monday, December 31, 1877, in the presence of three members of the institute, the liquefaction of atmospheric air was accomplished.

